# Early embryonic thermal programming and post-hatch flavonoid (*Scutellaria baicalensis*) supplementation enhanced immune response markers in broiler chickens

**DOI:** 10.3389/fvets.2025.1537116

**Published:** 2025-01-28

**Authors:** Sadid Al Amaz, Md Ahosanul Haque Shahid, Rajesh Jha, Birendra Mishra

**Affiliations:** Department of Human Nutrition, Food and Animal Sciences, College of Tropical Agriculture and Human Resources, University of Hawai’i at Manoa, Honolulu, HI, United States

**Keywords:** antibody, broilers, climate change, heat stress, immunity section: Animal nutrition and metabolism

## Abstract

**Introduction:**

Genetic selection in broiler chickens has led to increased muscle mass without comparable respiratory and cardiovascular system development, limiting the birds’ capacity to withstand high ambient temperatures and making them vulnerable to heat stress (HS). Early embryonic Thermal Manipulation (TM) has been suggested as an effective and sustainable way to mitigate the adverse effects of HS. This study investigated how these interventions influenced the immune status of broiler chickens exposed to HS.

**Methods:**

Cobb 500 fertile eggs (*n* = 600) were incubated according to guidelines. On embryonic day (ED) 12, the eggs were split into two groups: (1) Control, kept at standard temperature until hatch day (ED 21) and (2) Thermal Manipulation (TM), exposed to 38.5°C with 55% humidity for 12 h daily from ED 12 to ED 18. After hatching, chicks were divided into (1) Control, (2) TM, (3) Control under Heat Stress (CHS), (4) TM under Heat Stress (TMHS), (5) Control with Heat Stress and Supplementation (CHSS), and (6) TM with Heat Stress and Supplementation (TMHSS). For the first 21 days, all chicks were raised under normal conditions. From day 22 to day 35, groups CHS, TMHS, CHSS, and TMHSS experienced chronic heat stress (32–33°C for 8 h daily), while the Control and TM groups remained in a thermoneutral environment (22–24°C).

**Results and discussion:**

TM significantly increased (*p* < 0.05) *AvBD11, IL4,* and *TLR21* expression in the spleen. TM and baicalein supplementation significantly decreased (*p <* 0.05) *TLR15* expression. In the bursa, TM significantly increased (*p <* 0.05) *IL4* expression. The combination of TM with baicalein significantly increased (*p <* 0.05) *CD3* and decreased (*p <* 0.05) *TLR1* expression. Interestingly, TM alone significantly decreased (*p <* 0.05) *IFNg* expression under HS condition. In the thymus, TM significantly decreased (*p <* 0.05) *IL10* and *TLR15*, while incorporating baicalein with TM decreased (*p <* 0.05) *AvBD6* expression.

**Conclusion:**

TM improved the immune status of broiler chickens under normal conditions. When combined with baicalein, TM mitigated the negative effects of heat stress by boosting key immune-related gene expression in the spleen, bursa, and thymus.

## 1 Introduction

Global warming, caused by greenhouse gas emissions from 2011 to 2020, has increased the average global surface temperature by 1.1°C above the pre-industrial level of 1850–1900 (1). This has resulted in rapid changes in the biosphere, cryosphere, ocean, and atmosphere, leading to significant impacts on environmental, animal, and human life. Elevated temperatures may impede innate and adaptive immunity in chickens, leading to increased susceptibility to additional infections and diseases, contributing to the negative impact of heat stress (HS) (2). Stress-induced immunosuppression negatively impacts the immune system, leading to impaired immune organ cells and tissues, causing abnormal immune function and temporary or prolonged immune response dysfunction (3). Stress-induced immunosuppression in food animals, especially poultry, poses significant risks to food safety and public health.

The immune organs of birds can be divided into two groups: the central immune organs (thymus, bursa, and bone marrow) and the peripheral immune organs (spleen, cecum, and tonsil). Central immune organs develop from primordial cells during the embryonic stage and mature into fully functional organs as the bird ages. The spleen, the largest immune organ in poultry, plays a crucial role in regulating both humoral and cellular immunity. Stress-induced immunosuppression impacts immune-related genes in spleen tissues (4). The bursa of Fabricius is the primary lymphoid organ in birds, playing a crucial role in developing B-lymphocytes (5). Furthermore, the bursa contains immune-competent B-lymphocytes that can produce antibodies locally (6). The thymus is an essential immune organ responsible for developing and selecting naive T-lymphocytes. Chronic HS in broilers resulted in the atrophy of the thymus and other immune tissues (7). Heat stress during the embryonic stage can also impede thymus development in broiler chickens. Elevated temperatures may impact the thymus, potentially influencing the production of T-cells and, consequently, the immune capabilities mediated by the lymphocyte (8). Antibodies are proteins produced as part of the immune system’s response to an antigen (9). Chickens have three classes of immunoglobulins (IgA, IgM, and IgY) (10). It has been proposed that chickens also have antibodies similar to mammalian IgE and IgD (11). The molecular weights, morphology, and immunoelectrophoretic mobility of chicken IgA and IgM closely resemble those of mammalian IgA and IgM. Chicken IgY is transferred from the mother to the embryo through the egg yolk (12), resulting in elevated levels of chicken IgY in the egg yolk. Chicken IgY (or chicken IgG) is the functional counterpart to mammalian IgG in birds. However, it exhibits several functional differences compared to mammalian IgG (13).

Various strategies have been employed to mitigate the detrimental impacts of HS in poultry production. These approaches encompass genetic, managerial, and nutritional strategies, among others. The genetic strategy entailed developing poultry strains possessing specific genes, such as the naked neck, frizzle, and dwarf genes, which contribute to reducing HS (14, 15). The management strategies involve using appropriate housing design, provision of shade, use of sprinklers, implementation of cooling devices, and utilization of fans and ventilation systems (15). Nutritional interventions in poultry farming involve optimizing feed composition and supplementing essential micronutrients to improve the productivity of birds. The most frequently used methods to enhance production in the poultry industry involve using different supplements such as fat, antioxidants, yeast, and electrolytes (16). Enhancing embryonic thermal manipulation (TM) capacity to tolerate heat in poultry has been studied (17–19). In developmental biology, researchers have found that exposing embryos to higher incubation temperatures during important stages of their development can enhance their ability to tolerate high temperatures during the post-hatch period (20). This increased thermotolerance can be achieved through the embryos’ ability to respond quickly to thermal stress, adapt to higher temperatures through acclimation, and make epigenetic changes in response to temperature (21). Furthermore, evidence has firmly established that specific nutritional programming can effectively mitigate the adverse consequences of HS in broiler chickens. The root of *Scutellaria baicalensis* Georgi, commonly known as Huang Qin or Chinese skullcap, yields a primary flavonoid known as baicalein (5,6,7 trihydroxy flavone). Baicalein supplementation in the broiler’s diets resulted in enhanced growth, immune response, antioxidant profile, and serum lipid metabolism (22). Our previous study demonstrated that embryonic TM enhanced hatchability, thermotolerance capacity, and liver metabolism while reducing hatch time (23). In addition, pre-hatch embryonic TM and post-hatch baicalein supplementation enhanced body weight, average daily gain, average daily feed intake, feed conversion ratio, cecal microbial diversity, volatile fatty acids (24), liver metabolism, and muscle proliferation (25) of heat-stressed broilers.

Therefore, considering the effectiveness of pre-hatch TM and post-hatch baicalein supplementation on other growth performance and gut health variables, we hypothesized that pre-hatch TM and post-hatch baicalein supplementation would improve the immune status of heat-stressed broiler chickens. In this follow-up study, our objective was to examine the effects of TM and baicalein supplementation on plasma immunoglobulin (IgA and IgY) and the important immune markers in the spleen, bursa, and thymus in broiler chickens under HS conditions.

## 2 Materials and methods

### 2.1 Experimental design

This study employed animal experimentation and samples from our prior work (24). Briefly, we obtained 600 fertile Cobb 500 eggs from a local hatchery (Asagi Hatchery Inc., Honolulu, HI). Three incubators (GQF incubator, Savannah, GA) were used to randomly incubate the eggs. Each incubator contained 200 eggs. The eggs were kept at a standard temperature of 37.5°C with a relative humidity of 55% for 24 h/d for the first embryonic day (ED) 11. The eggs were examined using the candling technique to distinguish the viable embryos (*n* = 474) selected for the study. On ED 12, the eggs were split into two incubation groups: (1) Control (*n* = 236), which was kept at a standard temperature until the hatch day (ED 21) and (2) TM group (*n* = 238) which was kept at a temperature of 38.5°C with relative humidity (RH) of 55% for 12 h/d from ED 12 to ED 18, and then at a standard temperature from ED 19 to ED 21. Each treatment utilized two incubators equipped with automatic temperature regulation, 55% RH, and egg rotation every 2 h.

### 2.2 Hatching and rearing management

Upon hatching, unsexed day-old chicks (*n* = 360) were evenly distributed into two mains cohorts: the Control (*n* = 180 from the Control group at hatch) and the TM (*n* = 180 from the TM group at hatch). Subsequently, three treatment groups were formed from each of the Control and TM cohorts, resulting in six treatment groups. The post-hatch treatments comprised: (1) Control, (2) Control heat stress (CHS), (3) Control heat stress supplement (CHSS), (4) Thermal manipulation (TM), (5) Thermal manipulation heat stress (TMHS), and (6) Thermal manipulation heat stress supplement (TMHSS). After being individually weighed and having their wings tagged, the chicks were divided into 36 pens at random (10 birds per pen), resulting in 6 repetitions for each treatment group (*n* = 60 birds per treatment). The chicks were reared on a floor pen covered with litter, following the standard Cobb-500 guidelines for raising and managing broiler chickens. Between d 21 and 35, birds in the CHS, CHSS, TMHS, and TMHSS treatments underwent cyclic heat stress exposure. This entailed subjecting them to 33–35°C temperatures from 10:00 to 18:00 h (to simulate natural conditions) and 22–24°C at night with 55% RH. Meanwhile, the Control and TM groups were raised at standard room temperature (22–24°C) with 55% RH throughout the study. The birds were observed three times daily (in the morning, afternoon, and evening) to guarantee appropriate supervision and well-being. The pens were subjected to a completely randomized allocation in this study. Broiler chickens were raised with a standard lighting system (23 h light: 1 h dark).

### 2.3 Diets

The basal diets for the Cobb 500 broilers were formulated using corn and soybean meal. These diets were divided into two phases: starter (from d 1 to d 21) and finisher (from d 22 to d 35). The formulation of these diets fulfilled the nutritional needs of the broilers (26). Food and water were provided freely and without restriction throughout the study. The Control, CHS, TM, and TMHS groups were provided the basal diet for the entire study duration. The CHSS and TMHSS groups were provided basal diets supplemented with baicalein (250 mg/kg) for the entire study duration. The dosage of the baicalein supplement was selected based on its antioxidant properties and the dosage utilized in studies involving rodents, cattle, and humans. The composition and nutrient profile of diets are presented in Table 1.

**Table 1:** Composition of experimental diets and their nutrient profile.

Ingredients, %	Starter diet (1–21 days)		Finisher diet (22–35 days)	
	Control	Test	Control	Test
Corn	53.67	53.67	60.84	60.84
SBM	38.00	38.00	31.00	31.00
Soybean oil	5.00	5.00	5.50	5.50
Limestone	1.35	1.35	1.20	1.20
Monocalcium phosphate	0.75	0.75	0.44	0.44
Lysine	0.18	0.18	0.10	0.10
Met	0.18	0.18	0.13	0.13
Thr	0.04	0.04	0.00	0.00
Tryptophan	0.00	0.00	0.00	0.00
Choline Cl	0.00	0.00	0.00	0.00
Nacl	0.20	0.20	0.18	0.18
Sodium bicarbonate	0.12	0.12	0.10	0.10
Vitamin + mineral mix^a^	0.50	0.50	0.50	0.50
Baicalein	0.00	0.00020	0.00	0.00020
Phytase	0.01	0.01	0.01	0.01
Total	100.00	100.00	100.00	100.00
Nutrients contents in the diet %				
MEn, kcal/kg	3,040	3,040	3,165	3,165
CP	21.47	21.47	18.54	18.54
Ca	0.91	0.91	0.77	0.77
Total P	0.71	0.71	0.61	0.61
AvP	0.45	0.45	0.37	0.37
Lys	1.32	1.32	1.09	1.09
Met	0.52	0.52	0.44	0.44
Cys	0.42	0.42	0.40	0.40
Thr	0.87	0.87	0.73	0.73
Trp	0.31	0.31	0.27	0.27
Met + Cys	0.92	0.92	0.82	0.82
Arg	1.55	1.55	1.35	1.35
Val	1.18	1.18	1.05	1.05
Ile	0.90	0.90	0.78	0.78
Leu	1.82	1.82	1.66	1.66
NDF	8.86	8.86	8.73	8.73
CF	3.84	3.84	3.51	3.51
Na	0.16	0.16	0.14	0.14
Cl	0.16	0.16	0.15	0.15
Choline (mg/kg)	1,371	1,371	1,224	1,224
dig Lys%	1.17	1.17	0.95	0.95
dig Met%	0.48	0.48	0.40	0.40
dig Thr%	0.67	0.67	0.55	0.55

a Provides following nutrients (per kg of diet): vitamin A (trans-retinyl acetate), 10,000 IU; vitamin D_3_ (cholecalciferol), 3,000 IU; vitamin E (all-*rac*-tocopherol-acetate), 30 mg; vitamin B_1_, 2 mg; vitamin B_2_, 8 mg; vitamin B_6_, 4 mg; vitamin B_12_ (cyanocobalamin), 0.025 mg; vitamin K_3_ (bisulfate menadione complex), 3 mg; choline (choline chloride), 250 mg; nicotinic acid, 60 mg; pantothenic acid (D-calcium pantothenate), 15 mg; folic acid, 1.5 mg; betaíne anhydrous, 80 mg; D-biotin, 0.15 mg; zinc (ZnO), 80 mg; manganese (MnO), 70 mg iron (FeCO_3_), 60 mg; copper (CuSO_4_·5H_2_O), 8 mg; iodine (KI), 2 mg; selenium (Na_2_SeO_3_), 0.2 m.

### 2.4 Sample collection

At the end of the animal trial (d35), one bird from each pen (*n* = 6 per treatment) was euthanized using carbon dioxide asphyxiation for sampling. Spleen, bursa, and thymus tissues were collected, snap-frozen, and preserved at −80°C until RNA extraction. Blood samples were collected directly from the heart as soon as birds were euthanized and stored at −80°C.

### 2.5 Enzyme-linked immunosorbent assay

The chicken plasma immunoglobulins IgA and IgY levels were quantified using a commercial ELISA Kit (Bethyl Laboratories, Montgomery, TX) according to the manufacturer’s instructions. IgA was subjected to standard gradients with concentrations ranging from 1,000 ng/mL to 0 ng/mL, prepared through serial dilutions. Similarly, IgY was subjected to standard gradients with concentrations ranging from 500 ng/mL to 0 ng/mL, also prepared through serial dilutions. The plasma samples were diluted using Dilution Buffer B. IgA was diluted at ratios of 1:1,000 and 1:2,000, while IgY was diluted at 1:100,000 and 1:200,000. Afterward, 100 μL of each standard and sample was distributed into the wells of the ELISA plate, which had already been coated with anti-chicken antibodies in duplicate. The plate was placed in an environment with a constant temperature for 1 hour and subsequently cleaned. Subsequently, 100 μL of Chicken IgA or IgY Detection Antibody was introduced into each well, followed by an incubation period of 1 h and subsequent washing. The colorimetric reaction was accelerated for 30 min by introducing streptavidin-conjugated horseradish peroxidase along with TMB substrate. The reaction was halted by introducing 100 μL of Stop Solution, and the absorbance was quantified at 450 nm using a multimode ELISA plate reader (SynergyLX, Biotek, Santa Clara, CA).

### 2.6 Quantitative real-time PCR

The qPCR experiment was conducted in a 10 μL reaction mixture, including 3 μL cDNA and 7 μL PCR mix utilizing the Quant Studio™ 3 System (Applied Biosystems). The PCR mixture was generated using 5 μL of PowerUp SYBR Green Master Mix (Applied Biosystems) and 1 μL of both forward and reverse primers specific to the target gene. The PCR mixture and cDNA samples were placed into a 96-well optical plate and sealed with transparent optical adhesive films (Applied Biosystems), as previously documented (20). The specificity of each primer was confirmed using melting curve analysis. The expression of glyceraldehyde 3-phosphate dehydrogenase (GAPDH), beta-actin (*β*-actin), and TATA-Box Binding Protein (TBP) was examined in triplicate across the samples to identify the most stable housekeeping gene in endometrial tissues. GAPDH was the most consistent housekeeping gene. The target genes were analyzed in duplicates, and the expression levels were quantified using cycle threshold (Ct) values following normalization with GAPDH. The fold change for each gene was determined utilizing the comparative CT approach (2^−ΔΔCt^ method). The list of gene primers is presented in Supplementary Table S1.

### 2.7 Statistical analyses

The gene expression data were analyzed utilizing GraphPad software (GraphPad Software, San Diego, CA). After conducting a one-way analysis of variance (ANOVA), the Tukey-HSD test was employed to compare the means of the different treatment groups. The statistical significance threshold was established at a level of *p < 0.05*. The presentation of all data is as mean ± SEM.

## 3 Results

### 3.1 Effects of TM and post-hatch baicalein supplementation on the plasma immunoglobulins

**Figure 1:**
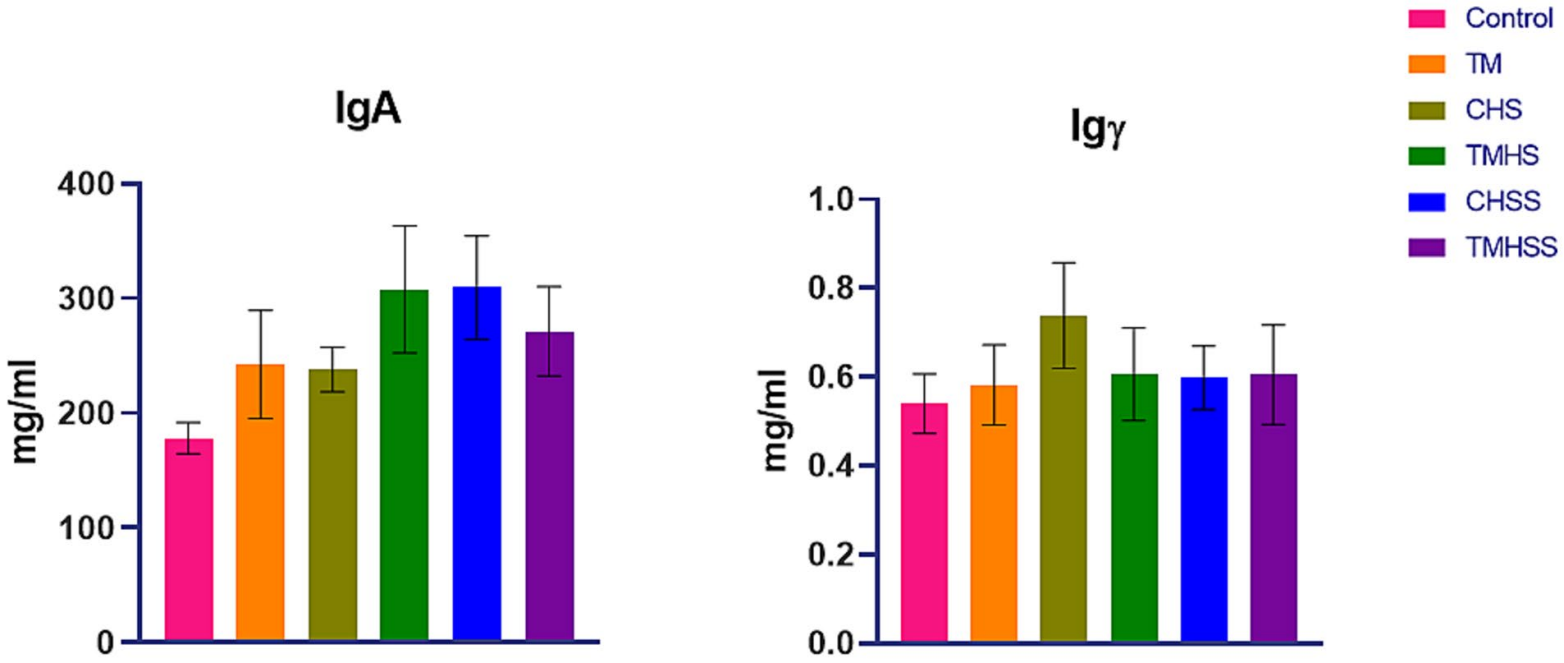
Effects of TM on plasma IgA and Igγ. Data presented as mean ± SEM. Different letters indicate a significant difference among the treatment groups.

Figure 1 shows the plasma immunoglobulin (IgA and IgY) concentration. There were no significant differences (*p < 0.05*) in IgA and IgY concentrations among the treatment groups.

### 3.2 Effects of TM and post-hatch baicalein supplementation on the spleen gene expression

**Figure 2:**
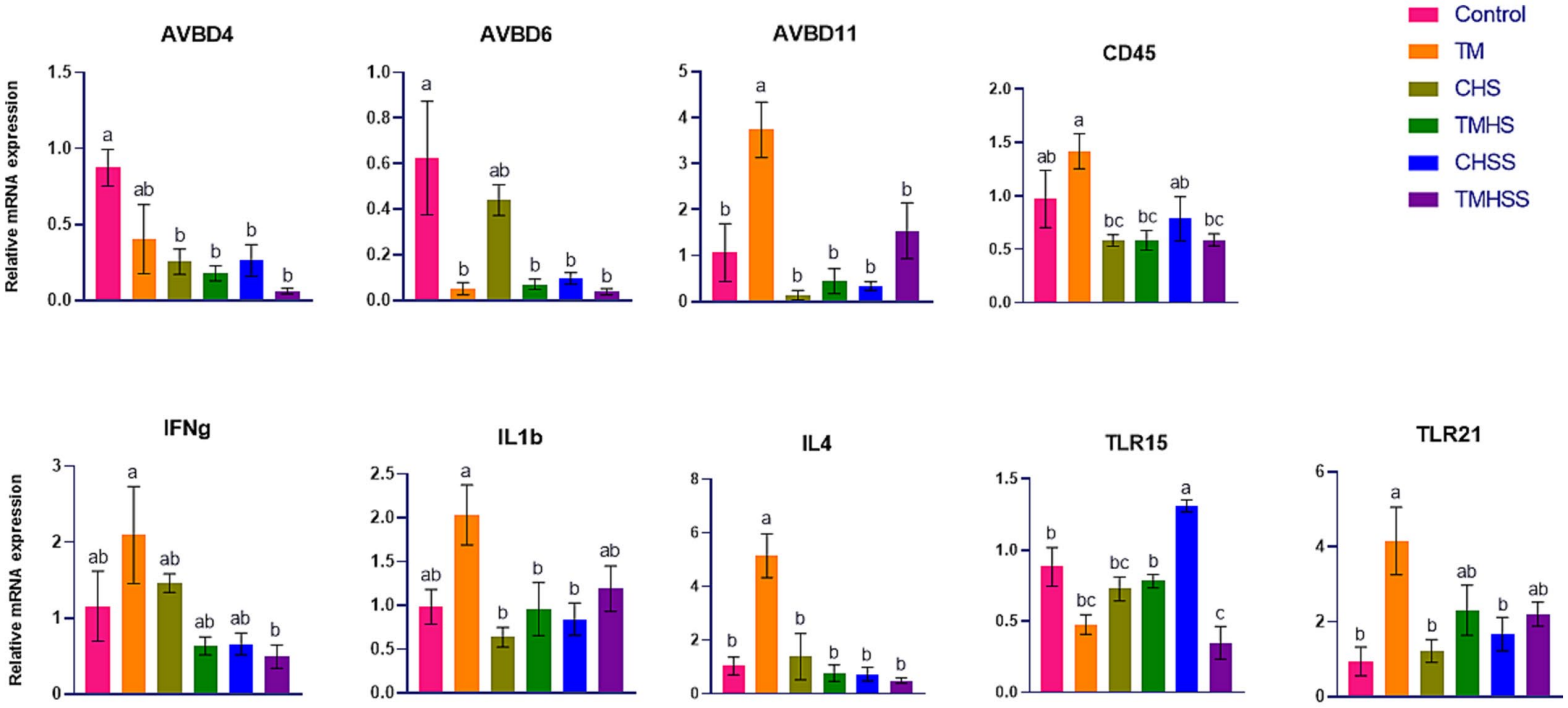
Effects of TM on the mRNA expression of immune-related genes in the spleen. Data presented as mean ± SEM. Different letters indicate a significant difference among the treatment groups.

The expression pattern of the immune-related genes (*AvBD4, AvBD6, AvBD11, CD45, IFNy, IL1b, IL4, TLR15,* and *TLR21*) among the treatments is shown in Figure 2. *AvBD4* expression was significantly higher (*p < 0.05*) in the Control group than in the CHS, TMHS, CHSS and TMHSS groups. *AvBD6* expression was significantly higher (*p < 0.05*) in the Control group than in the TM, TMHS, CHSS, and TMHSS groups. *AvBD11* and *IL4* expression were significantly higher (*p < 0.05*) in the TM group compared to the other treatment groups. *CD45* expression was significantly higher (*p < 0.05*) in the TM group compared to the CHS, TMHS, and TMHSS groups. *IFNg* expression was significantly higher (*p < 0.05*) in the TM group compared to the TMHSS group. *IL1 β* expression was significantly higher (*p < 0.05*) in the TM group compared to the CHS, TMHS, and CHSS groups. *TLR15* expression was significantly higher (*p < 0.05*) in the CHSS group than in other treatment groups. *TLR21* expression was significantly higher (*p < 0.05*) in the Control group than in the TM group compared to the Control, CHS, and CHSS groups.

### 3.3 Effects of TM and baicalein supplement on the bursa gene expression

**Figure 3:**
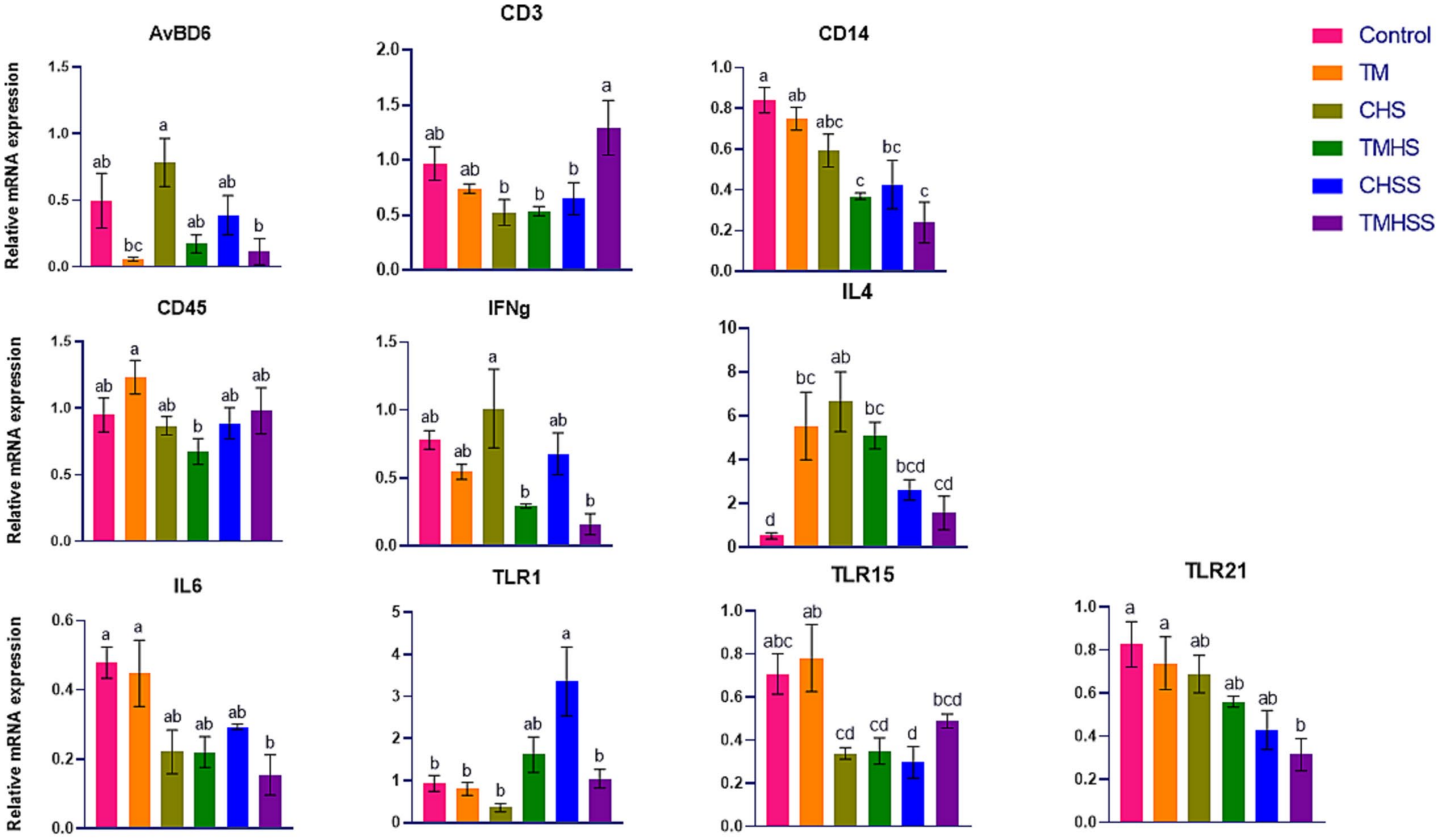
Effects of TM on the mRNA expression of immune-related genes in the bursa. Data presented as mean ± SEM. Different letters indicate a significant difference among the treatment groups.

The expression pattern of the immune-related genes (*AvBD6, CD14, CD45, IFNg, IL4, IL6, TLR1, TLR15,* and *TLR21*) among the treatments is shown in Figure 3. *AvBD6* expression was significantly higher (*p < 0.05*) in the CHS group than in the TM and TMHSS group. *CD3* expression was significantly higher (*p < 0.05*) in the TMHSS group than in the CHS, TMHS, and CHSS groups. *CD14* expression was significantly lower (*p < 0.05*) in the TMHS, CHSS, and TMHSS groups compared to the Control group. *CD45* expression was significantly lower (*p < 0.05*) in the TMHS group compared to the TM group. *IFNg* expression was significantly higher (*p < 0.05*) in the CHS group compared to the TMHS and TMHSS groups. *IL4* expression was significantly lower (*p < 0.05*) in the Control group compared to the TM, CHS, and TMHS groups. *IL6* expression was significantly lower (*p < 0.05*) in the TMHSS group compared to the Control and TM groups. *TLR1* expression was significantly higher (*p < 0.05*) in the CHSS group compared to the Control, TM, CHS, and TMHSS groups. *TLR15* expression was significantly higher (*p < 0.05*) in the TM group compared to the CHS, TMHS, and CHSS groups. *TLR21* expression was significantly lower (*p < 0.05*) in the TMHSS group compared to the Control and TM groups.

### 3.4 Effects of TM and baicalein supplement on the thymus gene expression

**Figure 4:**
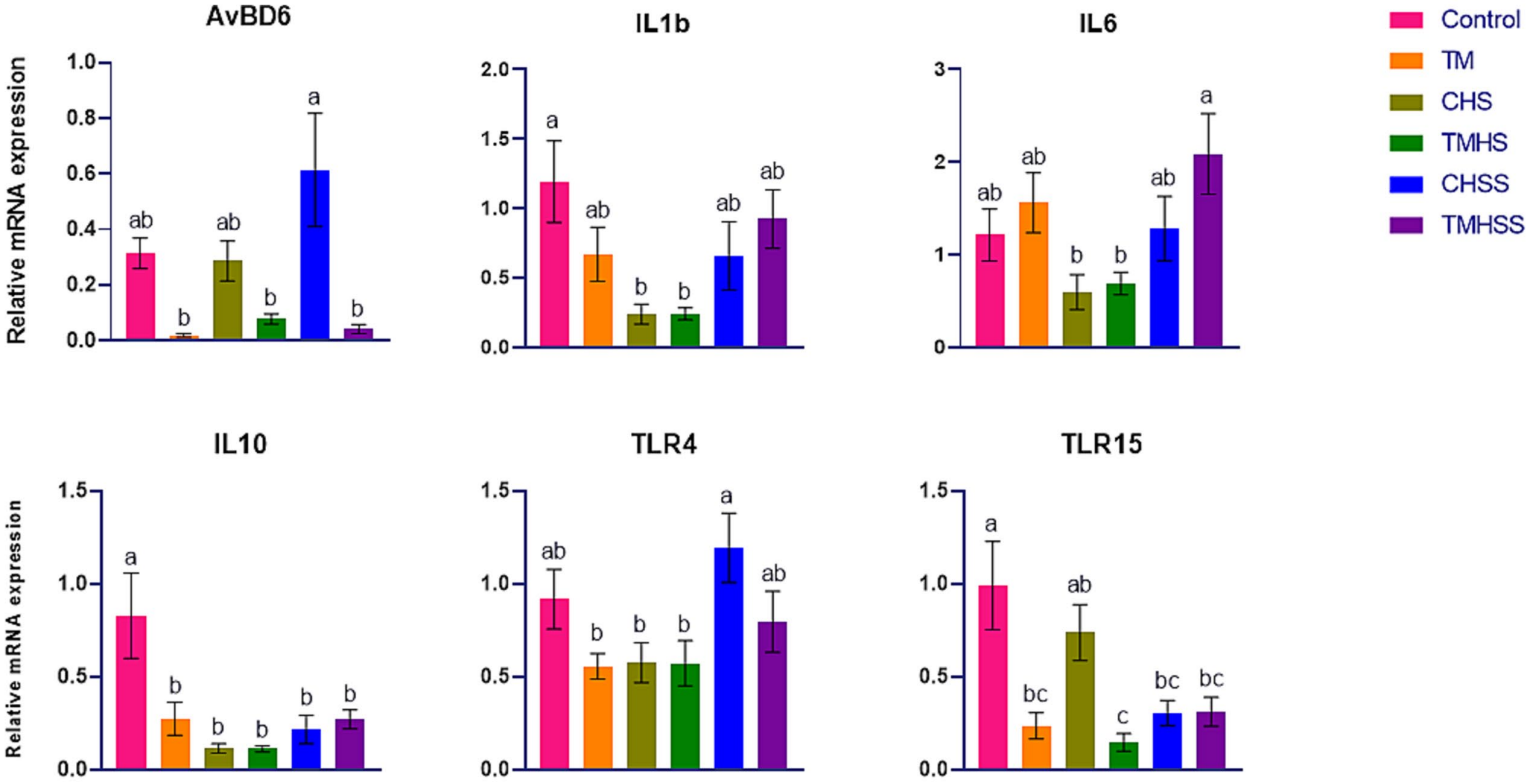
Effects of TM on the mRNA expression of immune-related genes in the thymus. Data presented as mean ± SEM. Different letters indicate a significant difference among the treatment groups.

The expression pattern of the immune-related genes (*AvBD4, IL1b, IL10, TLR4, and TLR15*) among the treatments is shown in Figure 4. *AvBD6* expression was significantly higher (*p < 0.05*) in the CHSS group than in the TM, TMHS, and TMHSS groups. *IL1β* expression was significantly higher (*p < 0.05*) in the Control group than in the CHS and TMHS groups. *IL6* expression was significantly higher (*p < 0.05*) in the TMHSS group than in the CHS and TMHS groups. *IL10* expression was significantly higher (*p < 0.05*) in the Control group compared to the other treatment groups. *TLR4* expression was significantly higher (*p < 0.05*) in the CHSS group than in the TM, CHS, and TMHS groups. *TLR15* expression was significantly higher (*p < 0.05*) in the Control group than in the TM, TMHS, CHSS, and TMHSS groups.

## 4 Discussion

Avian immune systems consist of humoral (antibody-mediated) and cellular (cell-mediated) defense mechanisms, similar to mammalian immune systems (27). The humoral immune system of chickens has been found to contain three classes of immunoglobulins, IgM, IgA, and IgY, homologs of mammalian IgM, IgA, and IgG, respectively (10). Plasma immunoglobulins can indicate the immune system’s condition in birds. Environmental factors like HS can impact the birds’ immune system and levels of circulating antibodies. TM, TMHS, and TM with baiclein supplement (TMHSS) did not affect plasma IgA and IgY levels in this study. More research is necessary to explain the effects of TM on HS on the immune physiology of birds, as there is a lack of scientific evidence.

The spleen acts as a hematopoietic organ in embryonic development. It plays a crucial role in immune responses, especially in response to blood-borne antigens, following the migration of lymphocytes and the formation of red and white pulps (28). In this study, we measured some important immune-related genes in the spleen; among them, *AvBD4, AvBD6, AvBD11, CD45, IFNg, IL1b, IL4, TLR15,* and *TLR21* were significantly different among the treatments. A class of antimicrobial peptides known as defensins is found in vertebrates, and it serves as the first line of defense against pathogens and immunological stimulants. In chickens, 14 defensins have been identified: avian *β*-defensin 1 to 14 (AvBD1-14) (29). *AvBD4* exhibits potent antibacterial and antifungal properties (30). In this study, significantly lower expression of *AvBD4* in the HS groups (CHS, TMHS, CHSS, and TMHSS) may suggest an adaptive response in the HS condition. *AvBD6* demonstrated robust antimicrobial properties against *Staphylococcus aureus*, *Salmonella typhimurium*, and *E. coli* in low-salinity environments. Compared to *AvBD12*, *AvBD6* is more capable of neutralizing lipopolysaccharides due to its larger net positive charge (31). *AvBD11* modulates innate immunity and exhibits various activities against bacterial pathogens, Eimeria parasites, and the avian influenza virus. Furthermore, it appears to regulate cell viability, proliferation, and invasiveness (32). A significantly higher *AvBD11* mRNA expression might have aided in decreasing the *AVBD6* mRNA expression significantly in the TM group. Higher *AvBD11* expression indicates higher innate immunity; this immune response may present a long-term defense and make a favorable immune condition for birds. That’s why there was a lower *AvBD6* response. *CD45* controls the communication between T cells and macrophages by interacting with its ligand, macrophage galactose-type lectin. This interaction occurs through binding to *CD45* N-acetyl galactosamine, causing a reduction in T cell proliferation and increasing proinflammatory cytokine production, ultimately resulting in T cell apoptosis (33). As a result, *CD45* is a crucial element in controlling innate immunity signaling. This study found a significantly higher *CD45* response in the TM group compared to the CHS, TMHS, and CHSS groups, which means TM with or without did not work during the HS condition. Interferon-gamma (*IFNg*) is a cytokine that regulates microbial infections. *IFNg* can induce tolerogenic effects on innate and adaptive immune cells, leading to tolerance in antigen-presenting cells and enhancing the function and development of regulatory T cells (34). *IFNg* also stimulates macrophages to enhance phagocytosis, tumoricidal abilities, and intracellular elimination of pathogens. It stimulates macrophages to produce various inflammatory mediators and reactive oxygen and nitrogen compounds. *IFNg* enhances the cellular immune response following infection and vaccination (35, 36). This study showed a significant decrease in *IFNg* gene expression in TMHSS compared to the TM group. This suggests a potential protective capacity of the baicalein supplementation in TM birds, even in elevated temperatures. Interleukin-1β (*IL1b*) is a crucial mediator of inflammation. It exacerbates damage during acute tissue injury and chronic disease and is necessary for the host response and resistance to pathogens (37). This study found a significantly higher *IL1b* in TM birds compared to CHS, TMHS and CHSS birds. Additionally, *IL1B* in the TMHSS birds was not affected by the HS, which indicates TM combined with baicalein supplement was more effective in HS condition than the TM alone. *IL4* is a cytokine that controls antibody production, hematopoiesis, inflammation, and the formation of effector T-cell responses. The substance is generated by a specific group of activated blood cells, such as T cells and Fc epsilon R1^+^ mast cells and basophils (38). A significantly higher *IL4* expression in TM compared to the Control group may show evidence of a higher immune response in the thermally manipulated bird. *TLR15* triggers the body’s natural defense system and detects secreted proteases from bacteria and fungi that are associated with causing disease (39). *TLR15* activation requires the cleavage of the receptor’s ectodomain and the stimulation of NF-kB-dependent gene transcription (40). This study found a significantly lower *TLR15* mRNA expression in the TMHSS group compared to the Control, TMHS and CHSS groups, further bolstering TM birds’ potent immunity under HS conditions. Toll-like receptor 21 (*TLR21*) is a non-mammalian receptor that identifies unmethylated CpG DNA and is seen as a functional equivalent of mammalian *TLR9*. *TLR21* might significantly impact how the body reacts to infections from various pathogens, such as bacteria, viruses, and RNA and DNA strands (41). *TLR21* gene expression was significantly higher in the spleen of the TM group than in the Control, CHS, and CHSS groups, which suggests a higher immune response in the body that fights against foreign pathogens. Taken together, the higher expression of immune-related genes in the TM and TMHSS indicates a higher immune response in the spleen of these birds, which robustly shows the effectiveness of TM and baicalein supplementation in both normal and HS conditions.

Like the spleen, we analyzed the same immune-related genes in the bursa. However, only *AvBD6, CD3, CD14, CD45, IFNg, IL4, IL6, TLR1, TLR15,* and *TLR21* were significantly different among the treatments. The CD3 antigen is a surface structure linked to the T-cell receptor (TCR) that creates a complex that plays a role in recognizing antigens and transmitting signals. The CD3-T cell receptor complex is crucial in the T-cell-mediated immune response as it recognizes antigens and transmits signals to activate T-lymphocytes (42). This study found a significantly higher *CD3* expression in the TMHSS group than the CHSS, CHS, and TMHS groups, suggesting the baicalein supplementation’s effectiveness in the thermally manipulated birds even though they were in HS condition. CD14 is a surface antigen that acts as a binding site for bacterial lipopolysaccharides (43). Gram-negative bacteria’s cell wall contains a major pathogen-associated molecular pattern (PAMP) called lipopolysaccharide (LPS), which is recognized by the TLR4 in mammals along with serum protein, lipopolysaccharide-binding protein (LBP), and the CD14 co-receptor. The activation and expression of innate immune genes in chicken heterophils are directly influenced by the engagement of LBP and CD14/TLR4 in LPS-mediated processes (44). Numerically lower expression of *AvBD6 and CD14* and numerically higher expression of *CD45* in the thermally manipulated groups (TM, TMHS, and TMHSS) may imply an improved immunity in those birds with or without HS. The TMHS and TMHSS groups showed significantly lower IFNg expression compared to the CHS group, while the TM group had higher IL4 expression than the Control group. This suggests that TM may help regulate T-cell responses and enhance phagocytosis, and the removal of intracellular pathogens. Both the CHS and TMHS groups exhibited significantly higher IL-4 expression than the control group, possibly because the birds were acclimatized to heat stress from day 22 to day 35. The role of Chicken IL6 in the pro-inflammatory response has been established. IL6 is a secreted protein that recruits and regulates cells in natural and acquired immune responses (45). IL6 assists in providing temporary defense against infection or harm by notifying the immune system about the origin of inflammation. It regulates the immune response by promoting the growth and specialization of white blood cells that eliminate harmful microorganisms (46). Interestingly, the *IL6* gene in chickens serves as a heat-shock gene. HSF3 activates the *IL6* in response to HS (47). In our previous study (24) with the same birds, we did not find any significant changes in the *HSF3* gene expression, and maybe that is why there was not much change in the *IL6* expression (except in the TMHSS group) in this study. It might be due to the higher antioxidant capacity of the baicalein supplement. Heterophils have a wide range of TLR expressions, which indicates their main function as the initial defense cells in birds against bacterial, viral, fungal, and parasitic infections (48). TLR1 plays a significant role in the recognition of triacyl lipopeptides. Macrophages lacking TLR1 mice exhibited reduced production of inflammatory cytokines when exposed to various types of triacyl lipopeptides and lipoproteins derived from mycobacteria (49). The TLR1 levels were significantly higher in the CHSS birds compared to the Control, TM, CHS, and TMHSS groups, indicating a need for further investigation. TLR15, which is unique to birds, has the ability to recognize a wide range of ligands derived from both viruses and bacteria (40). TLR21 is a pattern recognition receptor that detects microbial DNA to trigger the host’s infection response (50). This study found that *TLR15* expression was significantly lower in the CHSS group compared to both the Control and TM groups. Additionally, it revealed that *TLR21* expression was significantly reduced in the TMHSS group when compared to the Control and TM groups. These findings indicate that baicalein may serve as a beneficial feed supplement for poultry, even under heat stress conditions.

Unlike spleen and bursa, we also analyzed the same set of immune-related genes in the thymus, but only *AvBD6, IL1b, IL6, IL10, TLR4,* and *TLR15* demonstrated significant changes among the treatments. IL10 is a cytokine that has potent anti-inflammatory effects. It plays a crucial role in reducing the host’s immune response to pathogens, which helps protect the host from injury and maintain the normal balance of tissues. The dysregulation of IL10 is linked to heightened immunopathology following infection and an elevated susceptibility to various autoimmune diseases (51). Like mammalian species, chicken IL10 plays a significant role in determining the Th bias during *Eimeria* spp. infection. It inhibits the development of robust, IFNg-driven responses essential for controlling Eimeria infections (52). Another important immunoregulator during pathogen infection, including intracellular protozoa, is *IL10* (53). This study found a significant decrease in *IL10* expression in all groups compared to the Control group. It may indicate an adaptive response of the birds as the sample was collected after 2 weeks of HS. The role of TLR4 as the receptor for gram-negative lipopolysaccharide has been widely acknowledged. Furthermore, it attaches to endogenous molecules generated due to tissue damage. Therefore, TLR4 is a crucial receptor that brings together infectious and noninfectious stimuli to trigger an inflammatory response (54). The *TLR4* gene in chickens is also linked to the reaction to Salmonella (49). In this study, *AvBD6* was significantly lower in the TMHSS group than the CHSS group, which may indicate the higher immune response in the TM group, which further bolsters the benefits of baicalein supplementations during HS conditions. There is significantly higher *TLR4* expression in the CHSS group compared to the TM, CHS, and TMHS groups. Additionally, there is significantly higher *TLR15* expression in the Control group compared to the TM, CHS, CHSS, and TMHSS groups. These findings may also indicate an adaptive response in birds similar to IL-10.

## 5 Conclusion

Pre-hatch thermal manipulation and post-hatch baicalein supplementation did not affect the plasma immunoglobulins of broilers. However, TM alone improved the immune status in standard-raising conditions in the spleen and bursa. Interestingly, combining TM and baicalein significantly boosted the immunity under the HS condition. These phenomena may aid in regulating the immune genes of birds to an optimal level under high temperatures. As a result, both TM and post-hatch baicalein supplementation have been observed to enhance the adaptability of the birds during the period of HS.

## Data Availability

The original contributions presented in the study are included in the article/Supplementary material, further inquiries can be directed to the corresponding author/s.

## References

[1] Calvin K Dasgupta D Krinner G Mukherji A Thorne PW Trisos C. Climate change 2023: synthesis report. Contribution of working groups I, II and III to the sixth assessment report of the intergovernmental panel on climate change [Core writing team, H. Lee and J. Romero (eds.)]. Geneva, Switzerland: Intergovernmental Panel on Climate Change (IPCC) (2023).

[2] Lara LJ Rostagno MH. Impact of heat stress on poultry production. Animals. (2013) 3:356–69. doi: 10.3390/ani3020356, PMID: 26487407 PMC4494392

[3] Shini S Huff GR Shini A Kaiser P. Understanding stress-induced immunosuppression: exploration of cytokine and chemokine gene profiles in chicken peripheral leukocytes. Poult Sci. (2010) 89:841–51. doi: 10.3382/ps.2009-00483, PMID: 20308420

[4] Guo Y Su A Tian H Zhai M Li W Tian Y. Transcriptomic analysis of spleen revealed mechanism of dexamethasone-induced immune suppression in chicks. Genes. (2020) 11:513. doi: 10.3390/genes11050513, PMID: 32384708 PMC7288455

[5] Cooper MD Peterson RDA South MA Good RA. The FUNCTIONS of the THYMUS system and the BURSA system in the chicken. J Exp Med. (1966) 123:75–102. doi: 10.1084/jem.123.1.75, PMID: 5323079 PMC2138128

[6] Wu B Cui H Peng X Fang J Cui W Liu X. Pathology of bursae of Fabricius in methionine-deficient broiler chickens. Nutrients. (2013) 5:877–86. doi: 10.3390/nu5030877, PMID: 23486195 PMC3705324

[7] Quinteiro-Filho WM Ribeiro A Ferraz-de-Paula V Pinheiro ML Sakai M Sá LRM. Heat stress impairs performance parameters, induces intestinal injury, and decreases macrophage activity in broiler chickens. Poult Sci. (2010) 89:1905–14. doi: 10.3382/ps.2010-00812, PMID: 20709975

[8] Monson MS Van Goor AG Ashwell CM Persia ME Rothschild MF Schmidt CJ. Immunomodulatory effects of heat stress and lipopolysaccharide on the bursal transcriptome in two distinct chicken lines. BMC Genomics. (2018) 19:643. doi: 10.1186/s12864-018-5033-y, PMID: 30165812 PMC6117931

[9] Pereira EPV Van Tilburg MF Florean EOPT Guedes MIF. Egg yolk antibodies (IgY) and their applications in human and veterinary health: a review. Int Immunopharmacol. (2019) 73:293–303. doi: 10.1016/j.intimp.2019.05.015, PMID: 31128529 PMC7106195

[10] Leslie GACLW. Phylogeny of immunoglobulin structure and function: III immunoglobulins of the chicken. J Exp Med. (1969) 130:1337–52. doi: 10.1084/jem.130.6.1337, PMID: 5352783 PMC2138693

[11] Campbell RD Dodds AW Porter RR. The binding of human complement component C4 to antibody-antigen aggregates. Biochem J. (1980) 189:67–80. doi: 10.1042/bj1890067, PMID: 6906229 PMC1161918

[12] Murai A Hamano T Kakiuchi M Kobayashi M Horio F. Evaluation of a receptor gene responsible for maternal blood IgY transfer into egg yolks using bursectomized IgY-depleted chickens. Poult Sci. (2020) 99:1914–20. doi: 10.1016/j.psj.2019.11.045, PMID: 32241471 PMC7587843

[13] Carlander D Stålberg J Larsson A. Chicken antibodies: a clinical chemistry perspective. Ups J Med Sci. (1999) 104:179–89. doi: 10.3109/03009739909178961, PMID: 10680951

[14] Yunis R Cahaner A. The effects of the naked neck (Na) and frizzle (F) genes on growth and meat yield of broilers and their interactions with ambient temperatures and potential growth rate. Poult Sci. (1999) 78:1347–52. doi: 10.1093/ps/78.10.1347, PMID: 10536780

[15] Wasti S Sah N Mishra B. Impact of heat stress on poultry health and performances, and potential mitigation strategies. Animals. (2020) 10:1–19. doi: 10.3390/ani10081266, PMID: 32722335 PMC7460371

[16] Vandana GD Sejian V Lees AM Pragna P Silpa MV Maloney SK. Heat stress and poultry production: impact and amelioration. Int J Biometeorol. (2021) 65:163–79. doi: 10.1007/s00484-020-02023-7, PMID: 33025116

[17] Yahav S Collin A Shinder D Picard M. Thermal manipulations during broiler chick embryogenesis: effects of timing and temperature. Poult Sci. (2004) 83:1959–63. doi: 10.1093/ps/83.12.1959, PMID: 15615007

[18] Loyau T Berri C Bedrani L Métayer-Coustard S Praud C Duelos MJ. Thermal manipulation of the embryo modifies the physiology and body composition of broiler chickens reared in floor pens without affecting breast meat processing quality. J Anim Sci. (2013) 91:3674–85. doi: 10.2527/jas.2013-6445 23736053

[19] Al Amaz S Mishra B. Embryonic thermal manipulation: a potential strategy to mitigate heat stress in broiler chickens for sustainable poultry production. J Anim Sci Biotechnol. (2024) 15:75. doi: 10.1186/s40104-024-01028-1, PMID: 38831417 PMC11149204

[20] Chaudhary A Mishra P Amaz SA Mahato PL Das R Jha R. Dietary supplementation of microalgae mitigates the negative effects of heat stress in broilers. Poult Sci. (2023) 102:102958. doi: 10.1016/j.psj.2023.102958, PMID: 37540947 PMC10407898

[21] Yalçin S Çabuk M Bruggeman V Babacanoǧlu E Buyse J Decuypere E. Acclimation to heat during incubation. 1. Embryonic morphological traits, blood biochemistry, and hatching performance. Poult Sci. (2008) 87:1219–28. doi: 10.3382/ps.2007-00435, PMID: 18493014

[22] Zhou Y Mao S Zhou M. Effect of the flavonoid baicalein as a feed additive on the growth performance, immunity, and antioxidant capacity of broiler chickens. Poult Sci. (2019) 98:2790–9. doi: 10.3382/ps/pez071, PMID: 30778569

[23] Amaz SA Shahid MAH Chaudhary A Jha R Mishra B. Embryonic thermal manipulation reduces hatch time, increases hatchability, thermotolerance, and liver metabolism in broiler embryos. Poult Sci. (2024) 103:103527. doi: 10.1016/j.psj.2024.103527, PMID: 38412748 PMC10907853

[24] Al Amaz S Chaudhary A Mahato PL Jha R Mishra B. Pre-hatch thermal manipulation of embryos and post-hatch baicalein supplementation mitigated heat stress in broiler chickens. J Anim Sci Biotechnol. (2024) 15:8. doi: 10.1186/s40104-023-00966-6, PMID: 38246989 PMC10802028

[25] Amaz SA Shahid MAH Jha R Mishra B. Pre-hatch thermal manipulation of embryos and post-hatch baicaleien supplementation increased liver metabolism, and muscle proliferation in broiler chickens. Poult Sci. (2024) 103:104155. doi: 10.1016/j.psj.2024.104155, PMID: 39216265 PMC11402044

[26] Cobb Broiler Management Guide. (2012). Available at: www.cobb-vantress.com (Accessed September 02, 2024).

[27] Ulmer-Franco AM. Transfer of chicken immunoglobulin Y (IgY) from the hen to the Chick. Avian Biol Res. (2012) 5:81–7. doi: 10.3184/175815512X13350053184471

[28] Brendolan A Rosado MM Carsetti R Selleri L Dear TN. Development and function of the mammalian spleen. BioEssays. (2007) 29:166–77. doi: 10.1002/bies.20528 17226804

[29] Lyu W Zhang L Gong Y Wen X Xiao Y Yang H. Developmental and tissue patterns of the basal expression of chicken avian β-Defensins. Biomed Res Int. (2020) 2020:1–12. doi: 10.1155/2020/2567861, PMID: 33490238 PMC7787727

[30] Yacoub HA Elazzazy AM Abuzinadah OAH Al-Hejin AM Mahmoud MM Harakeh SM. Antimicrobial activities of chicken β-defensin (4 and 10) peptides against pathogenic bacteria and fungi. Front Cell Infect Microbiol. (2015) 5:36. doi: 10.3389/fcimb.2015.00036, PMID: 25941665 PMC4400880

[31] Yang M Zhang C Zhang X Zhang MZ Rottinghaus GE Zhang S. Structure-function analysis of avian β-defensin-6 and β-defensin-12: role of charge and disulfide bridges. BMC Microbiol. (2016) 16:210. doi: 10.1186/s12866-016-0828-y, PMID: 27613063 PMC5016922

[32] Guyot N Meudal H Trapp S Iochmann S Silvestre A Jousset G. Structure, function, and evolution of *Gga* -AvBD11, the archetype of the structural avian-double-β-defensin family. Proc Natl Acad Sci USA. (2020) 117:337–45. doi: 10.1073/pnas.1912941117, PMID: 31871151 PMC6955361

[33] Schuette V Embgenbroich M Ulas T Welz M Schulte-Schrepping J Draffehn AM. Mannose receptor induces T-cell tolerance via inhibition of CD45 and up-regulation of CTLA-4. Proc Natl Acad Sci USA. (2016) 113:10649–54. doi: 10.1073/pnas.1605885113, PMID: 27601670 PMC5035904

[34] Rožman P Švajger U. The tolerogenic role of IFN-γ. Cytokine Growth Factor Rev. (2018) 41:40–53. doi: 10.1016/j.cytogfr.2018.04.001, PMID: 29655565

[35] Santhakumar D Rubbenstroth D Martinez-Sobrido L Munir M. Avian interferons and their antiviral effectors. Front Immunol. (2017) 8:49. doi: 10.3389/fimmu.2017.00049, PMID: 28197148 PMC5281639

[36] Bagheri S Paudel S Wijewardana V Kangethe RT Cattoli G Hess M. Production of interferon gamma and interleukin 17A in chicken T-cell subpopulations hallmarks the stimulation with live, irradiated and killed avian pathogenic *Escherichia coli*. Dev Comp Immunol. (2022) 133:104408. doi: 10.1016/j.dci.2022.104408, PMID: 35390358

[37] Lopez-Castejon G Brough D. Understanding the mechanism of IL-1β secretion. Cytokine Growth Factor Rev. (2011) 22:189–95. doi: 10.1016/j.cytogfr.2011.10.001, PMID: 22019906 PMC3714593

[38] Brown MAHJ. Functions of IL-4 and control of its expression. Crit Rev Immunol. (1997) 17:1–32. doi: 10.1615/critrevimmunol.v17.i1.10, PMID: 9034722

[39] De Zoete MR Bouwman LI Keestra AM Van Putten JPM. Cleavage and activation of a toll-like receptor by microbial proteases. Proc Natl Acad Sci USA. (2011) 108:4968–73. doi: 10.1073/pnas.1018135108, PMID: 21383168 PMC3064367

[40] Boyd AC Peroval MY Hammond JA Prickett MD Young JR Smith AL. TLR15 is unique to avian and reptilian lineages and recognizes a yeast-derived agonist. J Immunol. (2012) 189:4930–8. doi: 10.4049/jimmunol.1101790, PMID: 23066147

[41] Li S Wang G Liu D Liu Q Hu G. Cloning and expression analysis of a toll-like receptor 21 (TLR21) gene from turbot, *Scophthalmus maximus*. Dev Comp Immunol. (2017) 73:163–8. doi: 10.1016/j.dci.2017.03.021, PMID: 28359672

[42] Yang H Parkhouse RME Wileman T. Monoclonal antibodies that identify the CD3 molecules expressed specifically at the surface of porcine γδ-T cells. Immunology. (2005) 115:189–96. doi: 10.1111/j.1365-2567.2005.02137.x, PMID: 15885124 PMC1782146

[43] Panaro MA Cianciulli A Gagliardi N Mitolo CI Acquafredda A Cavallo P. CD14 major role during lipopolysaccharide-induced inflammation in chick embryo cardiomyocytes. FEMS Immunol Med Microbiol. (2008) 53:35–45. doi: 10.1111/j.1574-695X.2008.00397.x, PMID: 18355291

[44] Kogut M He H Kaiser P. Lipopolysaccharide binding protein/CD14/TLR4-dependent recognition of Salmonella LPS induces the functional activation of chicken Heterophils and up-regulation of pro-inflammatory cytokine and chemokine gene expression in these cells. Anim Biotechnol. (2005) 16:165–81. doi: 10.1080/10495390500264896, PMID: 16335810

[45] Kaiser JT Clausen T Bourenkow GP Bartunik H-D Steinbacher S Huber R. Crystal structure of a NifS-like protein from *Thermotoga maritima*: implications for iron Sulphur cluster assembly. J Mol Biol. (2000) 297:451–64. doi: 10.1006/jmbi.2000.3581, PMID: 10715213

[46] Rodes L. Effect of probiotics Lactobacillus and Bifidobacterium on gut-derived lipopolysaccharides and inflammatory cytokines: an in vitro study using a human colonic microbiota model. J Microbiol Biotechnol. (2013) 23:518–26. doi: 10.4014/jmb.1205.05018, PMID: 23568206

[47] Prakasam R Fujimoto M Takii R Hayashida N Takaki E Tan K. Chicken *IL-6* is a heat-shock gene. FEBS Lett. (2013) 587:3541–7. doi: 10.1016/j.febslet.2013.09.012, PMID: 24055475

[48] Kogut M Iqbal M He H Philbin V Kaiser P Smith A. Expression and function of toll-like receptors in chicken heterophils. Dev Comp Immunol. (2005) 29:791–807. doi: 10.1016/j.dci.2005.02.002, PMID: 15936435

[49] Kannaki TR Reddy MR Shanmugam M Verma PC Sharma RP. Chicken toll-like receptors and their role in immunity. Worlds Poult Sci J. (2010) 66:727–38. doi: 10.1017/S0043933910000693

[50] Chuang Y-C Tseng J-C Yang J-X Liu Y-L Yeh D-W Lai C-Y. Toll-like receptor 21 of chicken and duck recognize a broad Array of Immunostimulatory CpG-oligodeoxynucleotide sequences. Vaccine. (2020) 8:639. doi: 10.3390/vaccines8040639, PMID: 33147756 PMC7712946

[51] Iyer SS Cheng G. Role of interleukin 10 transcriptional regulation in inflammation and autoimmune disease. Crit Rev Immunol. (2012) 32:23–63. doi: 10.1615/CritRevImmunol.v32.i1.30, PMID: 22428854 PMC3410706

[52] Rothwell L Young JR Zoorob R Whittaker CA Hesketh P Archer A. Cloning and characterization of chicken IL-10 and its role in the immune response to *Eimeria maxima*. J Immunol. (2004) 173:2675–82. doi: 10.4049/jimmunol.173.4.2675, PMID: 15294985

[53] Wilson EH Wille-Reece U Dzierszinski F Hunter CA. A critical role for IL-10 in limiting inflammation during toxoplasmic encephalitis. J Neuroimmunol. (2005) 165:63–74. doi: 10.1016/j.jneuroim.2005.04.018, PMID: 16005735

[54] Molteni M Gemma S Rossetti C. The role of toll-like receptor 4 in infectious and noninfectious inflammation. Mediat Inflamm. (2016) 2016:1–9. doi: 10.1155/2016/6978936, PMID: 27293318 PMC4887650

