# Supplementary material for: Early embryonic thermal programming and post-hatch flavonoid (Scutellaria baicalensis) supplementation enhanced immune response markers in broiler chickens
Source: Front Vet Sci. 2025 Jan 28;12:1537116. doi: 10.3389/fvets.2025.1537116 (PMC11810927; doi:10.3389/fvets.2025.1537116)
Supplement: Supplementary file 1 [file Table_1.docx]

**Supplementary Table 1:** List of primers used in qPCR.

| Gene | Accession No. | Primer sequence | Amplicon length |
| --- | --- | --- | --- |
| *TBP* | XM_025148547.3 | F: TAGCCCGATGATGCCGTAT | 147 |
|  |  | R: GTTCCCTGTGTCGCTTGC |  |
| *GAPDH* | NM_204305.2 | F: AGCTTACTGGAATGGCTTTCCG | 122 |
|  |  | R: ATCAGCAGCAGCCTTCACTACC |  |
| *B-Actin* | NM_205518.2 | F:GAG AAA TTG TGC GTG ACA TCA | 152 |
|  |  | R: CCT GAA CCT CTC ATT GCC A |  |
| *AVBD4* | NM 001001610.2 | F: TACCTGCTGCTGTCTGTCCT | 244 |
|  |  | R: AGTCCACTGCCACATGATCC |  |
| *AVBD6* | NM_001001193.1 | F: CTCCAGGGTGTTGCAGGTCAG | 188 |
|  |  | R: ATTTCCCAGGAGAAGCCAGTG |  |
| *AVBD11* | XM_046913334.1 | F: CAGAGACACCTCCCGTTGTG | 377 |
|  |  | R: CGTCGCCTCTAACGAATTGC |  |
| *CD45* | NM_204417 | F: TATTCTTGGTGTTCTTGATTGTTGTG | 120 |
|  |  | R: CTGCTACAAGGCTGATGACTTCA |  |
| *IFNy* | NM_205149.2 | F: TGAGCATTTGAACTGAGCCA | 240 |
|  |  | R: ATCCTTTTCTCATTTCTCTCTGTCC |  |
| *IL 1b* | NM_205064.1 | F: TGCCTGCAGAAGAAGCCTCG | 204 |
|  |  | R: GACGGGCTCAAAAACCTCCT |  |
| *IL4* | NM_001030693 | F: TGTGCCCACGCTGTGCTTACA | 155 |
|  |  | R: CTTGTGGCAGTGCTGGCTCTCC |  |
| *TLR 15* | NM_001398239.1 | F: CTCGGGGCCTTCACAGATTT | 344 |
|  |  | R: GATCCATCTCCAGGTCGCTG |  |
| *TLR 21* | XM_040707023.2 | F: ACCTGCTGACCGACCTCTAT | 466 |
|  |  | R: AGCATGTTCTGTGACAGCGA |  |
| *CD3* | NM_205512 | F: GGACGCTCCCACCATATCAG | 180 |
|  |  | R: TGTCCATCATTCCGCTCACC |  |
| *CD14* | NM_001139478.2 | F: TGGACGACTCCACCATTGAC | 132 |
|  |  | R: CCATCTCCTGCACCTGAGTG |  |
| *IL6* | NM_204628.2 | F: TTCACCGTGTGCGAGAACAGC | 80 |
|  |  | R: CAGCCGTCCTCCTCCGTCAC |  |
| *TLR1* | [NM_001081709.4](https://www.ncbi.nlm.nih.gov/entrez/viewer.fcgi?db=nucleotide&id=2099395557) | F: AAGCTTCCCGTGGAGTGATG | 96 |
|  |  | R: GAAACCCAAGGTGGAGGAGG |  |
| *IL10* | NM_001004414 | F: TGTCACCGCTTCTTCACCTG | 105 |
|  |  | R: CTCCCCCATGGCTTTGTAGA |  |
| *TLR4* | [NM_001030693.2](https://www.ncbi.nlm.nih.gov/entrez/viewer.fcgi?db=nucleotide&id=2099396323) | F: CCAAACACCACCCTGGACTT | 227 |
|  |  | R: AGCGACGTTAAGCCATGGAA |  |

F=Forward primer, R=Reverse primer
